# Recruiting and retaining GPs and patients in intervention studies: the DEPS-GP project as a case study

**DOI:** 10.1186/1471-2288-7-42

**Published:** 2007-09-18

**Authors:** Michelle K Williamson, Jane Pirkis, Jon J Pfaff, Orla Tyson, Moira Sim, Ngaire Kerse, Nicola T Lautenschlager, Nigel P Stocks, Osvaldo P Almeida

**Affiliations:** 1 Centre for Health Policy, Programs and Economics, School of Population Health, University of Melbourne, Australia; 2 WA Centre for Health and Ageing, School of Psychiatry and Clinical Neurosciences, University of Western Australia & Royal Perth Hospital, Australia; 3 School of Nursing, Midwifery and Postgraduate Medicine, Edith Cowan University, Australia; 4 Department of General Practice and Primary Health Care, University of Auckland, New Zealand; 5 Discipline of General Practice, University of Adelaide, Australia

## Abstract

**Background:**

Recruiting and retaining GPs for research can prove difficult, and may result in sub-optimal patient participation where GPs are required to recruit patients. Low participation rates may affect the validity of research.

This paper describes a multi-faceted approach to maximise participation of GPs and their patients in intervention studies, using an Australian randomised controlled trial of a depression/suicidality management intervention as a case study. The paper aims to outline experiences that may be of interest to others considering engaging GPs and/or their patients in primary care studies.

**Methods:**

A case study approach is used to describe strategies for: (a) recruiting GPs; (b) encouraging GPs to recruit patients to complete a postal questionnaire; and (c) encouraging GPs to recruit patients as part of a practice audit. Participant retention strategies are discussed in light of reasons for withdrawal.

**Results:**

The strategies described, led to the recruitment of a higher than expected number of GPs (n = 772). Three hundred and eighty three GPs (49.6%) followed through with the intent to participate by sending out a total of 77,820 postal questionnaires, 22,251 (28.6%) of which were returned. Three hundred and three GPs (37.0%) participated in the practice audit, which aimed to recruit 20 patients per participating GP (i.e., a total of 6,060 older adults). In total, 5,143 patients (84.9%) were represented in the audit.

**Conclusion:**

Inexpensive methods were chosen to identify and recruit GPs; these relied on an existing database, minor promotion and a letter of invitation. Anecdotally, participating GPs agreed to be involved because they had an interest in the topic, believed the study would not impinge too greatly on their time, and appreciated the professional recognition afforded by the Continuing Professional Development (CPD) points associated with study participation. The study team established a strong rapport with GPs and their reception staff, offered clear instructions, and were as flexible and helpful as possible to retain GP participants. Nonetheless, we experienced attrition due to GPs' competing demands, eligibility, personnel issues and the perceived impact of the study on patients. A summary of effective and ineffective methods for recruitment and retention is provided.

## Background

Intervention studies in the general practice setting are increasing in number, in recognition of the fact that such studies can provide knowledge that improves clinical care and the overall health of the population [1-5]. These studies require the participation of general practitioners (GPs), but recruiting and retaining these providers can be difficult, particularly when there is a need for long-term follow-up. In Australia, Silagy and Carson [1] found that only a quarter of GPs expressed interest in research, and Askew et al [4] reported that only 3% were involved in research activities at the time of the survey. This may seem surprising considering that the very nature of medicine is grounded within scientific research and the current emphasis on evidence-based practice. Various reasons have been posited for this low level of involvement, including the demands of clinical practice, structural issues such as a lack of protected time for non-clinical work activities and funding models of practice, as well as a lack of interest in research [1,3,4,6-10]. In addition, whenever participation involves the recruitment of patients, low response rates by GPs can result in sub-optimal patient participation. The magnitude of this problem is not fully known because researchers do not always report GP or patient participation rates [5]. It is likely, however, that recruitment and retention difficulties have an impact on the validity of such research in some cases [5-9]. Of note, Askew et al [4] found that only half of their respondents had ever recruited patients into research.

The current paper describes a multi-faceted approach to facilitate and promote participation in clinical research by GPs and their patients within the practice setting. This is in line with the call by Croughan [11] for researchers in this field to *"... provide an explicit and detailed description of our recruitment methods" (p 979); *stating that research is needed to advance this field. In this paper, we describe a large-scale Australian randomised controlled trial as a relevant case study. By way of context, Australia comprises eight states and territories and has a population of 21,000,000 which is served by 21,671 GPs [12]. These GPs usually operate from community practices, and are most commonly paid on a fee-for-service basis either by directly billing Medicare Australia (the body that administers the Medicare Benefits Schedule, Australia's universal health insurance scheme), or by billing the patient who can then obtain a partial rebate from Medicare Australia.

Divisions of General Practice (legally incorporated entities which draw their membership from the GPs in the geographically-defined area which they serve) provide a range of services for Australia's GPs, including advocacy, educational programs, and opportunities to work with other stakeholders on issues of common interest via specific projects.

The trial, known as the DEPS-GP project, is funded by the National Health and Medical Research Council and *beyondblue: the National Depression Initiative*, and is being conducted by investigators from eight universities across five Australian states (New South Wales, Victoria, Queensland, Western Australia and South Australia). It has ethics approval from the participating universities and from the Royal Australian College of General Practitioners (RACGP), the registration body for GPs in Australia. The study is designed to test an educational intervention aimed at increasing awareness about depression and suicidality in later life, with a particular emphasis on screening and management. Participating GPs have been randomly allocated according to a computer-generated list of random numbers into an intervention and a control group. Participating GPs have not been advised about their group membership. Intervention GPs receive a personalised education program (tailored to meet their specific requirements) and control GPs receive generic information about depression and suicide prevention. Patient outcomes are being measured by a postal questionnaire administered to all older patients of each participating GP at baseline, 12 months and 24 months, and designed to assess the prevalence of depression and suicidality among patients of participating practitioners. GP outcomes are being measured by a practice audit conducted at baseline and 24 months, in which GPs' assessments of 20 consecutive older patients' mental health status are compared with these patients' self-reported mental health status.

The DEPS-GP project is currently part-way through its three year data collection period. This paper outlines a critical reflection on the strategies and processes used in the recruitment and retention of GPs and patients during the baseline postal questionnaire and practice audit. The following sections pertain only to recruitment and retention and not the overall research project.

## Methods

### Overview

This section covers some of the strategies used in recruitment and retention of GPs and their patients to this point in the study. Specifically, the section describes the strategies used to: (a) recruit GPs to the study; (b) encourage them to recruit patients for the postal questionnaire; and (c) encourage them to recruit patients for the practice audit and participate in the audit themselves.

### Recruiting GPs to the study

We purchased and used the list of GPs held by the Australasian Medical Publishing Company Proprietary Limited as the base for our sampling frame. To ensure sufficient statistical power to detect expected effects, we aimed to recruit a sample of 480 GPs. Initially, we invited a stratified random sample of 4,900 GPs to participate, but only 225 agreed to do so. To counter this, we invited the remaining GPs on the database to take part, bringing the total to 19,046. This number is greater than the total number of GPs across the five study states, due to the fact that the database included the names of some non-GPs and contained some inaccuracies (e.g., names appearing twice).

The invitation letter included a document describing the project, ethics information and a consent form. It also explained that the project qualified for RACGP Continuing Professional Development (CPD) points, the Better Outcomes in Mental Health Care program CPD points and the Australian College of Rural and Remote Medicine Professional Development Program CPD points. As a requirement of vocational registration, GPs are required to participate in the RACGP's quality assurance and continuing medical education program by obtaining CPD points. Other programs offer similar points systems in order for GPs to be able to claim extra benefits from Medicare for services, but these are not compulsory. A brief practice survey was also included with the invitation and was designed to assess whether potential participants met various inclusion criteria. Specifically, the survey asked whether the GP worked at least four sessions or the equivalent of two days a week, had at least 50 patients aged 60+ who spoke English, and was not planning to retire or move practice within the next two years.

During the recruitment phase, details about the project were published in *Infonet*. This is a national bimonthly newsletter about primary health care practice, policy and research, published by Primary Health Care Research and Information Service (PHC RIS).

Research assistants were employed in each state as the primary means of communication between the research team and the participating GPs. During the recruitment phase, the research assistants' role was to answer any questions about involvement from potential participants, referring questions on to one of the chief investigators, if necessary. To ensure consistency of information, the research assistants and chief investigators worked from a study manual, which contained background literature, the study protocol, information on administrative procedures and all documents relating to the project.

### Encouraging GPs to recruit patients for the postal questionnaire

In order for the study team to administer the postal questionnaire, GPs were required to identify their regular patients aged 60 years or over. This typically involved generating a list of names and addresses from relevant electronic record systems. The process was sometimes more labour intensive in instances where records were not electronic or in shared practices where patients of one GP could not be distinguished from patients of another.

GPs were then given two choices regarding the mailout of the questionnaire. They could provide the above list to the study team, in which case the study team conducted the mailout. Alternatively, they could elect to conduct the mailout themselves, in which case the study team provided them with the requisite number of questionnaires in pre-prepared envelopes for them to address and post themselves. Irrespective of the mailout method, each patient received the questionnaire in an envelope which also contained a personalised cover letter from their GP, project information, a consent form and a reply-paid envelope addressed to the study team.

All participating GPs and their reception staff were provided with detailed instructions regarding the postal questionnaire. These were specifically designed to equip them to field any questions from patients involved in the postal questionnaire. A poster describing the study and providing contact information about local study staff was also displayed in the GPs' waiting rooms. The poster made it clear to patients that they had not been targeted to participate in the postal questionnaire (or the audit) for any reason other than their being aged 60+ and a patient of the participating GP.

The research assistants kept ongoing contact with the GPs and reception staff to maintain their commitment to the project. Much effort was made by the project team to facilitate participation in any way possible, such as personal visits to practices to help staff generate the list of eligible patients and/or assist with the mailout. Numerous telephone calls were made throughout the process. In Victoria, for example, GPs were phoned an average of four to five times (this figure includes calls where the GP was unavailable and a message was left). Those who elected to send out the questionnaire themselves required an average of five calls to ensure that the task was completed, whereas those who chose to let the study team send the questionnaire out on their behalf required an average of three calls.

GPs were not offered financial incentives at any time during the study. However, as a token of appreciation and in order to promote the retention of participants in the study, we posted book vouchers to GPs and their staff once the mailout had been completed successfully.

### Encouraging GPs to recruit patients as part of the practice audit

After completing the postal questionnaire stage, GPs were required to undertake a practice audit. This involved each GP (or, more commonly, his or her reception staff) recruiting 20 consecutive patients aged 60+ who attended the practice for any presenting problem during the audit period. Consenting patients were invited to complete a brief questionnaire assessing their mental health status prior to seeing their GP. The questionnaire was then placed in a sealed envelope. After seeing the patient, the GP completed a brief summary regarding the visit. The patient questionnaire and the GP summary form were then returned to the study team. The patient and the GP were each blind to what the other had written. Patients' names were not collected on either the patient questionnaire or the GP summary form; these were subsequently matched for analysis by sex and date of birth only.

All participating GPs were sent a package of materials and instructions which asked them to complete the practice audit within six to eight weeks of receipt. As with the postal questionnaire, detailed instructions and an information sheet were also provided to reception staff in order to equip them to field any questions from patients involved in the audit.

As with the postal questionnaire, the role of the research assistants during the practice audit involved energetically pursuing personal contact with participating GPs and their reception staff to keep them 'on board', and encouraging them to complete tasks within the prescribed time frame. Each research assistant kept consistent and thorough records of these contacts. In Victoria, for example, each GP was contacted by telephone three or four times on average (again, this figure includes occasions where the GP was unavailable and a message was left).

### Data analysis

Routinely-collected data on recruitment and retention of GPs were collated and analysed. Telephone logs were also coded and analysed, in order to determine the reasons for GPs withdrawing. There were some stylistic differences in record keeping between the different research assistants, but the logs were sufficiently thorough and coding categories were broad enough to allow for these differences. All data were analysed manually and are presented as simple frequencies and percentages.

## Results

The results reported here offer a numerical picture of the successes and limitations of the methods described above. The discussion section below provides further detail of these results and relates them to each method in turn, making inferences based on participants' reason for withdrawal.

### Initial GP response rates

**Figure 1:**
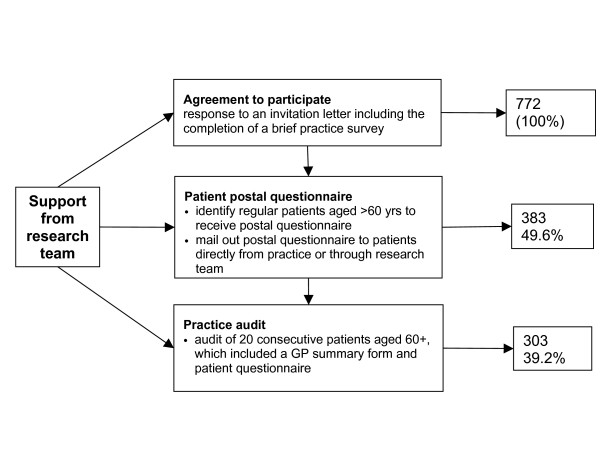
Summary of retention of general practitioner participants from recruitment through postal questionnaire and practice audit.

Figure 1 provides a summary of the number of GPs participating at each stage of the process and therefore the retention of participants at each stage. Table 1 shows a more detailed breakdown of participation by state. Our sampling and recruitment strategy yielded 772 GPs who indicated that they were willing to participate in the project. This exceeded our original aim of 480 GPs, and equated to an initial overall response rate of 4.1%. These figures underestimate the true response rates because, as previously noted, the denominators are inflated due to the over-inclusive nature of the database of practitioners.

**Table 1:** Response and retention rates of general practitioners according to Australian state

	**Target**	**Sampling frame**	**GPs initially agreeing to participate**		**GPs participating in postal questionnaire**		**GPs participating in practice audit**	
	
	**N**	**N**	**N**	**%**	**N**	**%**	**N**	**%**
**New South Wales**	168	6828	300	4.4	155	51.6	124	41.3
**Victoria**	120	4993	158	3.2	98	62.0	80	50.6
**Queensland**	72	3703	154	4.2	56	36.4	43	27.9
**Western Australia**	72	1849	99	5.4	49	49.5	38	38.4
**South Australia**	48	1673	61	3.6	25	41.0	18	29.5

**TOTAL**	480	19046	772	4.1	383	49.6	303	39.2

### Postal questionnaire response rate

Figure 1 and Table 1 show that 383 GPs participated in the postal questionnaire (49.6% of the initial participants). Participating GPs sent out a total of 77,820 questionnaires (that is, an average of 203 each). Two thirds of these (52,948) were sent directly by the GP, and one third (24,872) were sent by the study team, on the GP's behalf. In total, 22,251 questionnaires (28.6%) were returned, with slightly higher response rates achieved by the direct (15,775, or 29.8%) than indirect method (6,470, or 26.0%). These results do not take into account the number of questionnaires returned because the person was not known at the postal address or did not identify the corresponding GP as their current doctor, or were already deceased.

### Practice audit response rate

Figure 1 and Table 1 show that 303 of the initial participants took part in the practice audit (37.0%). In total, 5,143 patients were represented in the practice audit. Given that the 303 GPs who participated were asked to recruit 20 patients each for the practice audit (that is, a total of 6,060 patients), this equates to a response rate of 84.9%. At the end of the audit phase there were 157 and 146 GPs in the intervention and control groups respectively.

### GP reasons for withdrawal

Table 2 shows the reasons for GP withdrawal in each state. No reason was given or recorded for 45.4% of withdrawing GPs. Where a reason was provided, it was most commonly 'not enough time' or 'too busy'. This accounted for 29.2% of withdrawals overall (28.5% in the postal questionnaire and 32.5% in the practice audit). The next most common reason was 'not enough patients', which was the reason given by 7.2% of all GPs (8.0% in the postal questionnaire and 3.8% in the practice audit). 'Problems with creating patient list' required for the postal questionnaire was cited by 4.1% of GPs withdrawing during that stage and accounted for 4.9% of withdrawals overall. 'Patients didn't like questionnaire' was the next most common reason overall, given by 2.1% of all withdrawing GPs, and a particularly high proportion of those withdrawing at the practice audit stage (11.3%). Additional reasons were classified under the category of 'other' and accounted for 9.0% of all withdrawals. These included explanations such as 'overseas travel', 'health problems' or 'personal reasons not related to time'. 'Difficulty recruiting patients' for the practice audit was cited by 5.0% of GPs during the practice audit stage but only accounted for 0.9% overall.

**Table 2:** General practitioners' stated reasons for withdrawing from the study

**POSTAL QUESTIONNAIRE**												
	**New South Wales**		**Victoria**		**Queensland**		**South Australia**		**Western Australia**		**TOTAL**	

	N	%	N	%	N	%	N	%	N	%	N	%

Not enough time/Too busy	37	25.5	23	38.3	35	35.7	9	18.0	7	19.4	111	28.5
Not enough patients	13	9.0	7	11.7	7	7.1	3	6.0	1	2.8	31	8.0
Difficulty recruiting patients	0	0.0	0	0.0	0	0.0	0	0.0	0	0.0	0	0.0
Patients didn't like questions/questionnaire	0	0.0	1	1.7	0	0.0	0	0.0	0	0.0	1	0.3
No staff support	2	1.4	1	1.7	0	0.0	0	0.0	1	2.8	4	1.0
No colleague support	1	0.7	1	1.7	1	1.0	1	2.0	1	2.8	5	1.3
Problems with creating patient list	11	7.6	3	5.0	1	1.0	1	2.0	3	8.3	19	4.9
Other	13	9.0	8	13.3	11	11.2	5	10.0	5	13.9	42	10.8
No reason	68	46.9	16	26.7	43	43.9	31	62.0	18	50.0	176	45.2

**Total**	**145**	**100.0**	**60**	**100.0**	**98**	**100.0**	**50**	**100.0**	**36**	**100.0**	**389**	**100.0**

**PRACTICE AUDIT**												

	**New South Wales**		**Victoria**		**Queensland**		**South Australia**		**Western Australia**		**TOTAL**	

	N	%	N	%	N	%	N	%	N	%	N	%

Not enough time/Too busy	11	35.5	9	50.0	3	23.1	0	0.0	3	42.9	26	32.5
Not enough patients	0	0.0	1	5.6	2	15.4	0	0.0	0	0.0	3	3.8
Difficulty recruiting patients	0	0.0	0	0.0	1	7.7	2	18.2	1	14.3	4	5.0
Patients didn't like questions/questionnaire	2	6.5	1	5.6	3	23.1	1	9.1	2	28.6	9	11.3
No staff support	0	0.0	0	0.0	0	0.0	0	0.0	0	0.0	0	0.0
No colleague support	0	0.0	0	0.0	1	7.7	0	0.0	0	0.0	1	1.3
Problems with creating patient list	0	0.0	0	0.0	0	0.0	0	0.0	0	0.0	0	0.0
Other	0	0.0	0	0.0	0	0.0	0	0.0	0	0.0	0	0.0
No reason	18	58.1	7	38.9	3	23.1	8	72.7	1	14.3	37	46.3

**Total**	**31**	**100.0**	**18**	**100.0**	**13**	**100.0**	**11**	**100.0**	**7**	**100.0**	**80**	**100.0**

**TOTAL**												

	**New South Wales**		**Victoria**		**Queensland**		**South Australia**		**Western Australia**		**TOTAL**	

	N	%	N	%	N	%	N	%	N	%	N	%

Not enough time/Too busy	48	27.3	32	41.0	38	34.2	9	14.8	10	23.3	137	29.2
Not enough patients	13	7.4	8	10.3	9	8.1	3	4.9	1	2.3	34	7.2
Difficulty recruiting patients	0	0.0	0	0.0	1	0.9	2	3.3	1	2.3	4	0.9
Patients didn't like questions/questionnaire	2	1.1	2	2.6	3	2.7	1	1.6	2	4.7	10	2.1
No staff support	2	1.1	1	1.3	0	0.0	0	0.0	1	2.3	4	0.9
No colleague support	1	0.6	1	1.3	2	1.8	1	1.6	1	2.3	6	1.3
Problems with creating patient list	11	6.3	3	3.8	1	0.9	1	1.6	3	7.0	19	4.1
Other	13	7.4	8	10.3	11	9.9	5	8.2	5	11.6	42	9.0
No reason	86	48.9	23	29.5	46	41.4	39	63.9	19	44.2	213	45.4

**Total**	**176**	**100.0**	**78**	**100.0**	**111**	**100.0**	**61**	**100.0**	**43**	**100.0**	**469**	**100.0**

## Discussion

The DEPS-GP project has been successful in recruiting a large sample of GPs and their older patients. However, the retention rate of participants to date highlights the challenges of maintaining GPs' participation and commitment. Critical reflection on the recruitment processes, strategies to maintain participation, and the reasons given by the GP at the time of withdrawal provides valuable insight to other researchers experiencing the challenges of recruiting within the general practice setting. Various aspects of these processes, strategies and reasons are considered in turn below, and a summary of these can be found in Table 3.

**Table 3:** Summary of barriers and enablers to the recruitment and retention of general practitioners

	**Enablers**	**Barriers**
**Recruitment**	• Use of existing database • Minor promotion • Letter of invitation • Appealing topic • Time commitment presented as minimal • CPD points	• Errors in database

**Retention**	• Establishing relationship with GP and clinic staff and providing regular contact • Minimising tasks for participants and providing support • Providing clear instructions for participation • Creating instructions on how to use software to complete specific project tasks	• GP overestimating eligibility • Project time-line changes • Tasks involved with the audit needed to be completed by GP rather than staff • Inability to follow study protocols • Use of non-standardised medical record system • Non-computerised practice • Patients not wanting to participate

Recruitment relied on the use of an existing database to identify the sampling frame. There may have been several alternative approaches. One approach would have been to use an alternative database, but the options were limited. For example, the 119 Divisions of General Practice in Australia each hold information about their own networks of GPs and have local knowledge that assists in maintaining the accuracy of their lists, but these data are not aggregated nationally so drawing on Divisional data was impractical [13]. Another approach would have been to generate our own list of GPs and/or to actively recruit GPs through our own networks, but this would have been labour-intensive and impractical given our required sample size, and would have potentially introduced sampling bias [6,9]. Veitch et al [8] have cautioned against using existing databases for recruitment because of their inherent inaccuracies, and it is true that the Australasian Medical Publishing Company Proprietary Limited database proved to be somewhat over-inclusive. However, we felt that the advantages of this approach outweighed the disadvantages. It resulted in a high absolute number of recruited GPs (n = 772) but a low overall response rate (4.1%).

Having identified the sampling frame, we pursued a recruitment strategy that involved minor promotion through *Infonet *and a letter of invitation, both of which have been cited in the literature as successful recruitment methods [2,3,8,14]. Our large sample size target dictated our choice; these methods were practical and less costly and labour-intensive than others that have been described in the literature, including telephone contact [2,3,5,8,14], practice visits by the study team [2,3,5,8], and the use of physician and/or peer recruiters [6,14].

Although we did not explicitly collect data on why our study GPs chose to participate, some inferences can be made. Studies that have looked at reasons for participation and non-participation have consistently found an interest in the research topic, minimal time commitment and professional recognition as influential [1,6]. Anecdotally, a number of our participants indicated that they had a particular interest in mental health and/or that they were keen to avail themselves of the CPD points available. Some also questioned the time commitment, and agreed to participate when they were satisfied that it was not too onerous. It is interesting that the highest response rate was recorded in Western Australia where the study originated. The investigators in this state may be known to the general practice population due to a previous study [15]. Victoria was the most difficult state to recruit from and this may be due to the high volume of research undertaken in general practice there.

Once GPs agreed to participate, establishing a relationship with them and their reception staff was crucial, as were providing clear instructions and maintaining regular contact and support. Frequent calls were made to check on progress and provide encouragement. Often the GPs had varying preferences with regard to communication, with some preferring fax or email communication over telephone contact and vice versa, and some choosing ad hoc contact and others preferring to set specific times for teleconferences. As it was not always possible to speak to the GP directly, the reception staff became an important point of contact. Our research assistants were dedicated to establishing rapport with both GPs and reception staff, providing them with clear instructions and protocols, and 'fitting in' with their stated preferences.

In addition, we endeavoured to make the tasks required of the GPs and reception staff as simple as possible. For example, a number of GPs had difficulty generating lists of patients eligible to receive the postal questionnaire (either directly or via the study team). Consistent with technological barriers identified in other studies [5], this was largely due to lack of knowledge of the relevant computerised system. We produced a step-by-step guide on how to interrogate standard databases in a manner that produced listings of patients in the relevant age group. In addition, research assistants visited practices to assist with the generation of lists and other aspects of the questionnaire process, such as sticking labels on envelopes. In most cases, this overcame technological problems, but in some instances (e.g., where the GP used a non-standard computerised medical records system, or worked in a non-computerised practice) residual difficulties remained.

The slightly higher response rate of questionnaires sent directly by the GP (29.8%) compared to the response rate of those posted by the research team on behalf of the GP (26.0%) suggests that a direct method would be preferable in future studies. This would have to be weighed against the extra time required by the research assistant in ensuring the GP completed the mailout within project timeframes and whether or not the GP would have participated had the indirect method not been offered. As recommended by Edwards et al [16], we included the covering letter from the GP and reply paid envelope and used coloured ink for the survey to increase the response rate of the questionnaire.

Analysis of telephone logs from each state showed the reasons for withdrawal stated in Table 2. In addition, informal feedback from participating GPs suggests that the above strategies were successful in keeping them 'on board'. Nonetheless, we acknowledge that our retention rates were worse than our initial recruitment rates, and that there were barriers to ongoing participation that we were not always able to address successfully.

Most notably, and consistent with other studies [2,17], withdrawing GPs reported that they were unable to complete the required tasks in the time available due to competing demands that were sometimes unforseen when they 'signed up'. This was particularly the case with the practice audit. Tasks associated with the practice audit created a greater burden for the GP than tasks associated with the postal questionnaire because the latter could often be completed by reception staff. Some GPs reported being too busy to turn their attention to the audit, and did not manage to open the package of audit materials; others were unable to find the time to set the audit up; and still others began the task, but found that their schedules were too hectic to allow them to identify their allotted 20 patients and conduct the audit with them. The time issues associated with the audit were exacerbated by the fact that the audit was delayed by 3 months, so some GPs who had anticipated that it would occur at a time of year when their load was relatively light found that it actually took place when they were particularly busy.

Some GPs were 'lost' to the study by virtue of ineligibility, despite the study team's best efforts to provide clear instructions regarding eligibility at the outset. Some GPs were found to be ineligible once the project began because it transpired that they had over-estimated the numbers of English-speaking patients aged 60 or over attending their practice. Others ruled themselves out by not adhering to study protocols (e.g., handing out questionnaires to patients when they presented for a consultation, rather than posting them out).

Further retention difficulties arose because of issues with personnel in the given GP's practice. In some instances reception staff acted as 'gate keepers', making it difficult for the study team to establish and maintain contact with the GP directly. In other cases, lack of available reception staff was a problem, particularly for GPs in solo practices. In addition, non-participating GPs in the participating GP's practice directly or indirectly raised barriers either by actively objecting to their colleague's participation, or because the shared patient record system rendered it impossible to identify patients of the participating GP.

In a small minority of cases, GPs withdrew during the postal questionnaire stage because they were concerned the project could have a negative impact on some of their patients. In one case, the situation arose because recipients of the postal questionnaire (or their carers) raised concerns about the content of the questionnaire (specifically the items related to suicidality). This GP felt that continued participation would interfere with their practice and therefore withdrew.

## Conclusion

To summarise, we believe that our experience with recruitment and retention of GPs in the DEPS-GP project can provide valuable lessons for future projects. We chose inexpensive methods to identify and recruit GPs, relying on an existing database, minor promotion and a letter of invitation, and succeeded in recruiting a substantial sample of GPs. Anecdotally, our participating GPs agreed to be involved because they had an interest in the topic, believed the study would not impinge too greatly on their time, and appreciated the professional recognition afforded by the CPD points associated with study participation. Our study team was dedicated to retaining these GPs in the study, establishing a strong rapport with them and their reception staff, offering them clear instructions, and being as flexible and helpful as possible. Nonetheless, our retention rates were not as good as our recruitment rates, and we experienced attrition due to reasons of competing demands, eligibility, personnel issues and the perceived impact of the study on patients. We would recommend that strategies to maximise recruitment and retention, such as those found under the heading 'enablers' in Table 3, be given prominent consideration in future GP research.

## Competing interests

The author(s) declare that they have no competing interests.

## Authors' contributions

MKW and JP originated the idea for this paper and led the drafting of the manuscript. JP, JJP, OT, MS, NK, NTL, NPS, and OPA contributed to the conception and drafting of the manuscript. JP, JJP, NK, NTL, MS, NPS and OPA are leading investigators of the DEPS-GP Project. All authors read and approved the final manuscript.

## Pre-publication history

The pre-publication history for this paper can be accessed here:


